# Xenodiagnosis in the wild: A methodology to investigate infectiousness for tick-borne bacteria in a songbird reservoir

**DOI:** 10.1016/j.crpvbd.2024.100210

**Published:** 2024-08-23

**Authors:** Jens Zarka, Dieter Heylen, Hein Sprong, Manoj Fonville, Joris Elst, Erik Matthysen

**Affiliations:** a Evolutionary Ecology Group, Department of Biology, University of Antwerp, 2610, Wilrijk, Belgium; b Centre for Infectious Diseases Research, National Institute for Public Health and the Environment, 3720 BA Bilthoven, the Netherlands

**Keywords:** Xenodiagnosis, Infectiousness, *Borrelia*, *Parus*, *Ixodes*

## Abstract

A crucial factor to predict the persistence and spread of infections in natural systems is the capacity of reservoir hosts to maintain the infection and transmit it to others. This is known to greatly vary within and between species and through time, although the latter part of the variation is often less well understood in the wild. *Borrelia garinii* is one of the causal agents of Lyme disease in humans and is transmitted among avian hosts by the hard tick *Ixodes ricinus*. Great tits are known to be a reservoir in Europe for *B. garinii*. For tick-borne pathogens like *B. garinii*, infectiousness or host-to-vector transmission can be measured using xenodiagnosis where pathogen-free vectors are fed on a host, and the blood-fed vectors are subsequently tested for the pathogen. Here we describe and evaluate a methodology to quantify infectiousness for tick-borne pathogens in individual wild great tits (*Parus major*), involving captures and recaptures of targeted individuals. The methodology can potentially be applied to other species where recapturing is sufficiently guaranteed. We successfully recaptured most of the infested great tits two to three days after initial infestation (i.e. just before ticks have fully fed) with sufficient numbers of *I. ricinus* larval ticks, which were subsequently screened for *B. garinii* using a newly developed *B. garinii-*specific real-time PCR assay. Higher larval tick numbers were recovered from birds during the breeding seasons than during the winter months. Our novel *B. garinii-*qPCR performed well, and greatly reduced the amount of Sanger sequencing needed. Preliminary results suggest both seasonal and individual variation in infectiousness; heterogeneity that needs to be unravelled to further understand the contribution of resident birds to the epidemiology of *B. garinii*.

## Highlights

• We conducted the first xenodiagnoses on a wild free-living host for any vector-borne disease.
• We successfully recaptured most great tits (*Parus major*) infested with larval ticks 2–3 days after infestation.
• We successfully screened ticks with our *B. garinii*-qPCR, greatly reducing the number of larvae that had to be sequenced.
• We discovered large seasonal variation in the ability of birds to transmit *B. garinii* to feeding *I. ricinus* larvae.

## Graphical abstract

**Figure:**
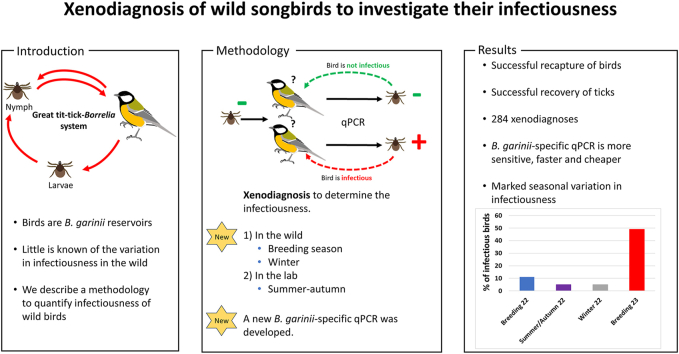

## 1 Introduction

Understanding how the dynamics of pathogen transmission contribute to spatio-temporal variation in endemic disease foci is of great importance, both from a fundamental ecological and evolutionary point of view, as well as for dealing with the epidemiological risk associated with zoonotic diseases.

Vector-borne disease systems are complex because of their multiple transmission pathways combined with dynamics of infections within hosts and vectors. The complexity intensifies when multiple hosts and vectors are involved, such as in tick-borne Lyme borreliosis, one of the most widespread zoonotic infections caused by the bacteria *Borrelia burgdorferi* (*sensu lato*) (*s.l*.) (Hubálek and Halouzka, 1997; Masuzawa, 2004; Faccini-Martínez et al., 2022). A competent host or vector species must be able to acquire and maintain the spirochetes for an extended period of time, as well as transmit them (Hanincová et al., 2008; Haven et al., 2012; Heylen et al., 2017a).

Many studies have focused on the competence of different host species, vectors and pathogen heterogeneity (Braks et al., 2016; O’Keeffe et al., 2020; Zinck et al., 2022). Additionally, numerous laboratory-based studies have investigated within host species heterogeneity (Humair et al., 1999; Hanincová et al., 2008; Zinck et al., 2023). However, far less emphasis has been placed on within host species heterogeneity in wild populations. This heterogeneity is expressed in individual variation in infectiousness defined as the ability of individuals of a competent species to infect other susceptible individuals. Heterogeneity of infectiousness can be partitioned into intrinsic individual variation (e.g. sex or immune status variation) (Råberg et al., 2007; Dunn et al., 2011; Cornelius et al., 2014), temporal changes within individuals, and spatiotemporal variation in population composition (e.g. due to age structure or seasonal migrations) (Becker et al., 2020). Even though heterogeneity in infectiousness within host species is well documented in many natural populations, the underlying causes for this are not well understood (Galvani and May 2005; Gopinath et al., 2014).

Studies on tick-borne infections, notably *Borrelia burgdorferi* (*s.l.*), typically focus on when and how hosts get infected by vectors. This includes many studies about pathogen loads in vectors and hosts (Jacquet et al., 2017; Zinck et al., 2022), host-vector contact rates (Estrada-Peña et al., 2019), and vector-to-host transmission of pathogens (Piesman et al., 1987; Eisen, 2020; Genné et al., 2023). In the case of *Borrelia burgdorferi* (*s.l.*) there is considerable variation among both host and vector species in their competence for specific genospecies and even strains (Hanincová et al., 2008; Heylen et al., 2014b; Wolcott et al., 2021). For example, particular *Borrelia* genospecies, such as *B. garinii*, are predominantly transmitted by birds to the common vector *Ixodes ricinus* (Heylen et al., 2013, 2014a). In contrast, very few studies have focused on when and how vectors are infected by reservoir hosts in the wild, and thus on variation in host-to-tick transmission or infectiousness within and between hosts. This is especially relevant in the Lyme borreliosis system, which lacks vertical transmission and has limited vector-to-vector transmission through co-feeding (Heylen et al., 2014a; Voordouw, 2015). Since infectiousness patterns are a critical variable to estimate the basic reproduction number (R_0_) of tick-borne infections, and in turn, R_0_ determines the ability of a tick-borne infection to spread and persist in nature, transmission models (Harrison et al., 2011; Nguyen et al., 2019) could be considerably improved by including parameters describing these patterns.

*Borrelia* detection within a host has been proven to be challenging. Although *Borrelia* transmission from host to ticks occurs in the skin, skin biopsies may underestimate host infectiousness (Norte et al., 2022). Especially early infections of birds may remain undetected by means of skin biopsies due to low abundance of *Borrelia* within these tissues (Norte et al., 2022). In contrast, ear biopsies of rodents have proven to be successful in determining infection status and tissue spirochete loads in the host (Råberg, 2012; Tschirren et al., 2013). Similarly low abundances of *Borrelia* within blood samples may cause *Borrelia* to be undetected (Norte et al., 2013a, 2022). An alternative to detection in blood or tissues is to analyse *Borrelia* in larvae feeding on their host, given that unfed larvae are mostly pathogen-free (Hanincová et al., 2003; Lommano et al., 2014; Barbour et al., 2015; but see Van Duijvendijk et al., 2016). However, since transmission from an infected host to a vector is not always assured, this method requires a minimum number of feeding larvae per host which reduces its efficiency and may also induce bias, especially if parasite loads are low and/or if they vary with environmental or individual characteristics (Heylen et al., 2013c; Klaus et al., 2016).

An alternative and more effective detection method that can be used is xenodiagnosis (Brumpt, 1914; Norte et al., 2022). In this method pathogen-free vectors are fed on a host (of known or unknown infection status), by means of active infestation, and the blood-fed vectors are subsequently tested for the pathogen of interest (Campos et al., 2007; Molina et al., 2012; Jiménez et al., 2014; Saavedra et al., 2016; Singh et al., 2020). Xenodiagnosis provides information on infectiousness or host-to-vector transmission, which is a critical variable for estimating R_0_ and the ability of the pathogen to invade and persist in an ecosystem. Unlike the other detection methods, xenodiagnosis directly and quantitatively measures the pathogen transmission of a potential reservoir to a vector. Negative results therefore do not necessarily indicate the absence of a pathogen in the host, but rather no or low infectiousness of the host. In *Borrelia* spp. research, xenodiagnosis has been important for determining host-to-tick transmission patterns over time and the discovery that different *Borrelia* genospecies are adapted to different reservoir hosts (Humair and Gern, 2000; Richter et al., 2004; Hanincová et al., 2008; Norte et al., 2013b). In addition, it has been used in experimental settings to assess the effects of antibiotic treatments on *Borrelia* infections within rodents (Bockenstedt et al., 2002; Hodzic et al., 2008) and the role of birds as *Borrelia* reservoir hosts (Norte et al., 2013b, 2020; Heylen et al., 2017a). More generally, xenodiagnosis is usually conducted in controlled experiments (Richter et al., 2004; Hanincová et al., 2008; Norte et al., 2013b) and very rarely for monitoring infectiousness in wild populations (but see Molina et al., 2012; Jiménez et al., 2014 for transmission of leishmaniasis by wild lagomorphs). To our knowledge, xenodiagnosis has never been used to monitor *Borrelia* infections in free-living animals, nor has it been used to monitor heterogeneity of infectiousness among hosts for any vector-borne infection.

Here we describe the first results of a study where we conducted xenodiagnoses on great tits *Parus major* to monitor infectiousness for *Borrelia garinii* (Heylen et al., 2013c, 2014a). Despite *B. garinii* causing Lyme borreliosis in humans, its ecology remains poorly understood (Mysterud et al., 2019b; Mtierova et al., 2020). Great tits are small non-migratory passerines and are one of Europe’s most abundant reservoir hosts of *B. garinii* (Heylen et al., 2013). Due to their high (re)capture rates, xenodiagnoses in the wild are possible on a large scale throughout most of the year. To conduct large-scale xenodiagnoses, it is of importance to screen the xenodiagnostic larvae quickly and efficiently. Therefore, we developed a *B. garinii-*specific real-time PCR. This enables us to increase detectability of *B. garinii* in ticks by decreasing the sequencing needed. In addition, it facilitates *B. garinii* detection in ticks co-infected with multiple *Borrelia* genospecies (Mysterud et al., 2019a). With this study, we present the first results of our methodology and discuss the success of the xenodiagnoses conducted in the wild.

## 2 Materials and methods

### 2.1 Capture and recapture of birds

Great tits *Parus major* are small cavity-breeding passerines known to use nestboxes when available in woodlands. Great tits were captured throughout the year (April 2022 to June 2023) in two separate areas “Peerdsbos” and “Boshoek” in Northern Belgium (Matthysen et al., 2001). The “Boshoek” plots are small forest fragments (6–12 ha), located in the south of Antwerp city (51°08′N, 4°32′E), surrounded by mainly agricultural lands and/or residential areas. The “Peerdsbos” plot is part of a larger (> 200 ha) forest-parkland complex at the northern edge of the city Antwerp (51°16′N, 4°30′E). Since the introduction of nestboxes in the forest plots in 1993, yearly population data have been collected in both areas. Nestboxes are regularly checked during the breeding season to record data on first-egg dates, clutch size and number of fledglings. Most parents are caught at the nestbox when feeding their young of approximately 8 days old (*c.*80–90% of all breeding birds). On the 14th or 15th day post-hatching, all chicks are ringed and measured. During summer and early autumn, birds are captured with mist nets at feeders, while during late autumn and winter, birds are caught inside the nestboxes while roosting. Previous research in both areas has shown that great tits are frequently infected with *B. burgdorferi* (*s.l.*), with *B. garinii* being the predominant genospecies in these birds (Heylen et al., 2013c; Heylen, 2016). Approximately 17.3% of *I. ricinus* larvae removed from great tits tested positive for *B. burgdorferi* (*s.l.*), but due to small sample sizes the exact prevalence of *B. garinii* cannot be precisely quantified (Heylen et al., 2013c).

In this study, birds were caught during three seasons: breeding season (mid-April to mid-June), summer/autumn (end-July to November) and winter (November to March). No birds were captured for xenodiagnoses during the nest building and egg laying time, from March to mid-April, as well as after the breeding season from mid-June to the end of July. In the breeding season and winter, we conducted xenodiagnoses in the field, while in summer/autumn, the birds were brought into the laboratory for the duration of the xenodiagnosis.

### 2.2 Xenodiagnosis

For all xenodiagnoses, birds were infested by placing 40 pathogen-free *I. ricinus* larvae from a laboratory colony (IS Insect Services GmbH, Berlin, Germany) on each bird. The larvae were carefully placed under the head feathers of the birds (Richter et al., 2000; Heylen and Matthysen, 2008). After placing the ticks on the birds, they were released back into the wild (unless if xenodiagnosis was performed in the laboratory, captivity conditions are described below). Birds were subsequently recaptured (or inspected in the laboratory) and ticks were removed before they could naturally detach from the host which usually occurs between 72 and 96 h after initiation of feeding (Heylen and Matthysen, 2010). During all seasons, except winter, before infesting the birds with xenodiagnostic ticks, we recorded the number of “wild” ticks already feeding on the birds and their life stages (larva or nymph; no adult *I. ricinus* ticks were found), which we did not remove. In winter, we did not perform these counts as we tried to reduce the handling time to increase recapture success of the birds. Furthermore, cooler winter temperatures reduce tick activity, and few if any wild ticks were expected on the birds before infestation (Gray, 2008; Heylen et al., 2014c; Kahl and Gray, 2023). Since our xenodiagnostic larvae are indistinguishable from wild ones, these pre-infestation counts gave us an estimate of the percentage of wild larvae among the collected larvae at the end of the xenodiagnosis. Minor differences in protocol per season are described in the following paragraphs.

In the breeding seasons of 2022 and 2023, adult great tits were captured, between 8:00 and 14:00 h, in their nestboxes when their young were 8–9 days old, as part of the routine population monitoring (Matthysen et al., 2001). They were infested with larvae and kept in a cotton bag for 1 h to increase the attachment success of the ticks (Heylen and Matthysen, 2008; Müller et al., 2013; Heylen et al., 2014a), before being released again. After releasing the birds, the cotton bags were checked for ticks that did not attach. During the first breeding season, birds were initially recaptured after three days. Due to the low tick recovery, we decided to recapture birds after two days which significantly increased tick recovery, suggesting that many larvae already detached within 72 h. We therefore continued recapturing the birds approximately 48 h after infestation. All ticks were removed, including wild ticks, and blood samples were taken. The blood samples, from the breeding season and the following seasons, were taken for the purpose of another study.

In the summer and autumn of 2022, we captured birds at a feeder with a mist net. Because of the low chance of recapturing individual birds at feeders within a fixed amount of time, the xenodiagnoses were conducted in the laboratory. Before being released in the indoor animal facility, the birds were infested in the field and kept in cotton bags for an hour, including transportation time to the animal facility. The bags were later checked for ticks that did not attach. Birds were kept in cages (80 × 41 × 50 cm) with unlimited food and water and artificial light corresponding to the outside day-night cycle. Approximately 48 h later, as in the breeding season, all ticks were recovered and blood samples were taken before releasing the birds back into the wild.

During the winter months of 2022–2023, we captured great tits roosting in nestboxes after sunset. Birds were infested by placing ticks under the head feathers but were immediately put back in the nestbox. Unlike in the other seasons, we did not keep the birds in a cotton bag to avoid unnecessary stress which might have reduced their likelihood of returning to roost in the same or other nestboxes and hence be recaptured (Tyller et al., 2012). Furthermore, we assumed that birds would quickly resume their resting position and there was no need to immobilize them to enhance tick attachment. Before sunrise, approximately 60 h later, we checked all nestboxes in the surrounding area to find the infested birds, followed by removal of ticks and taking blood samples. The birds were recaptured in the morning instead of the evening as the process of removing ticks and taking blood samples is time consuming and stressful for the birds. Therefore, to do this at the start of the day allows the birds to immediately start foraging and recovering from possible capture and handling stress.

### 2.3 PCR-based screening of *Borrelia* in ticks

Detection of *B. garinii* within ticks is conventionally done by performing a *B. burgdorferi* (*s.l*.)-qPCR followed by IGS-based genotyping of the *B. burgdorferi* (*s.l*.)-positive ticks to determine the *Borrelia* genospecies (Heylen et al., 2013c, 2014a). Since the success rate of IGS-based genotyping can vary considerably and is costly, we developed a *B. garinii-*specific qPCR to reduce genotyping efforts. We added the *B. garinii-*qPCR in between the two steps of the conventional approach. A detailed description is found in the following paragraphs.

DNA was extracted from all larval ticks using the Qiagen DNeasy Blood & Tissue Kit according to the manufacturer’s instructions (Qiagen, Hilden, Germany). Carrier-RNA was added to larvae that were not fully engorged to ensure a good DNA extraction. To check if the DNA was extracted correctly, a mitochondrial *16S* rRNA qPCR was performed on each tick (Schwaiger and Cassinotti, 2003).

**Fig. 1:**
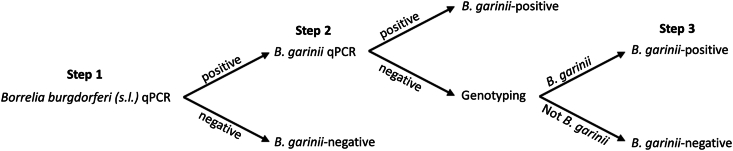
Flowchart of the new protocol to identify the presence of *B. garinii* in xenodiagnostic ticks. The old protocol consisted of Step 1 followed by Step 3 on the positive samples.

Following the DNA extraction, a real-time PCR was performed to identify the presence of *Borrelia burgdorferi* (*s.l.*) in the ticks using multiplex *flaB* and *ospA* (Fig. 1, Step 1). For the *flaB* gene, the forward primer 5′-CAG AIA GAG GTT CTA TAC AIA TTG AIA TAG A-3′, reverse primer 5′-GTG CAT TTG GTT AIA TTG YGC-3′ and an LNA probe Atto520-CAA CTI ACA GAI GAA AXT AAI AGA ATT GCT GAI CA containing an internal BHQ1 (X = T-BHQ1) was used. For the *ospA* gene, the forward primer 5′-AAT ATT TAT TGG GAA TAG GTC TAA-3′, reverse primer 5′-CTT TGT CTT TTT CTT TRC TTA CA-3′, and an LNA probe Atto520-AAG CAA AAT GTT AGC AGC CTT GA containing an internal BHQ1 (X = T-BHQ1) was used. The real-time PCR was performed in a Lightcycler 480 thermal cycler (Applied Biosystems, Waltham, MA, USA). Each reaction contained 4.5 μl of DNA extract (∼7.5% of tick lysate), 2 pmol of each primer and 0.075 pmol of the probe, 10 μl of 2× IQ multiplex Powermix (Bio-Rad, Hercules, CA, USA) and PCR-grade water to give a total of 20 μl. The program consisted of 5 min at 95 °C followed by 55 cycles (5 s at 94 °C and 35 s at 60 °C). Positive and negative controls, the latter being PCR-grade water, were used in duplicate on each plate of the qPCR run.

In case a tick tested positive for *B. burgdorferi* (*s.l.*), a real-time PCR for identifying *B. garinii* was performed (Fig. 1, Step 2). We developed a new real-time PCR, targeting the 5S-23S ribosomal DNA intergenic spacer region, using a new primer set to for identifying the presence of *B. garinii* in *I. ricinus* ticks using the forward primer 5′-TCT TTG TTT AAT CCA TGT CAA TAT A-3′, reverse primer 5′-AGG GTT TTT CTT TTA TAC TTT AAA-5′ and an LNA probe Atto425-AAY ATR AAY AT + C + TAA AAA + CAX AAA AAA + TAA AAT-C-pho containing an internal BHQ1 (X = T-BHQ1). The real-time PCR was performed in a Lightcycler 480 thermal cycler (Applied Biosystems). Each reaction contained 5 or 7 μl of DNA extract (∼10% of tick lysate), 2 pmol of each primer and 0.075 pmol of the probe, 10 μl of 2× IQ multiplex Powermix (Bio-Rad) and PCR-grade water to give a total of 20 μl. The program consisted of 5 min at 95 °C followed by 55 cycles (5 s at 94 °C and 35 s at 60 °C). Positive and negative controls, the latter being PCR-grade water, were used in duplicate on each plate of the qPCR run.

Analysis of all qPCRs were performed using the second derivative calculations for crossing point (Cp) values. Curves were assessed visually. A qPCR was considered positive when the Cp-values were < 40 and the amplification curves were sigmoid-shaped.

Whenever a tick tested negative with the *B. garinii* specific real-time PCR, IGS-based genotyping was performed to identify the *Borrelia* genospecies present (Fig. 1, Step 3) (Coipan et al., 2013; Van Gestel et al., 2024). This occasionally led to the detection of *B. garinii*-positive ticks that previously tested negative with the *B. garinii-*qPCR.

All larvae from the first breeding season were tested with the *Borrelia burgdorferi* (*s.l*.) test. On all *Borrelia burgdorferi* (*s.l.*)-positive larvae, we conducted both the *B. garinii*-specific test as well as IGS-based sequencing. This was done to compare the performance of the newly developed *B. garinii-*specific real-time PCR test to the conventional approach.

### 2.4 Data analysis

To evaluate fine-scale temporal and seasonal differences in the tick recovery success (i.e. proportion of larvae feeding on the birds at second capture over the total placed on the birds at first capture), we compared the tick recovery between 2 days and 3 days after placement of the ticks on the birds, as well as the pairwise contrasts between seasons. A Welch’s *t*-test was conducted to investigate the difference in the number of recovered larvae between 2- and 3-days post-infestation during the breeding season of 2022. Because the assumption for normality was not met, the square-root of the number of larvae was used. Seasonal variation in tick recovery was investigated using a Kruskal-Wallis test, since the assumption for normality was not met, followed by a Dunnʼs test. For this comparison we excluded birds recaptured after 3 days in the first season.

As a first exploration of seasonal variation in the proportion of birds with at least one *B. garinii*-positive larva, we performed a chi-square test. Again, the xenodiagnostic results from the birds recaptured after 3 days during breeding season 2022 were excluded from the analysis. This was because longer attachment time of the tick may have increased the probability of acquisition of *Borrelia* by the larval ticks, as demonstrated for *B. burgdorferi* (*sensu stricto*) (*s.s*.) in *I. scapularis* (Couret et al., 2017). However, due to the low prevalence of *B. garinii* in the first breeding season, we cannot test this specific hypothesis. A *post-hoc* analysis was conducted using the Bonferroni adjustment for multiple comparisons. All tests were conducted in R 4.0.3.

## 3 Results

### 3.1 Number of birds infested and recaptured

Over four consecutive seasons (breeding season 2022, summer/autumn 2022, winter 2022–2023, and breeding season 2023), we performed 398 infestations with larval ticks on great tits. Of those, 284 (71%) birds were recaptured (including xenodiagnoses in captivity) and feeding larvae were removed. In total, 241 different individuals underwent a full xenodiagnosis (i.e. larvae removed for screening), most of these birds (*n* = 197) were infested and recaptured a single time, while 39 and 3 individuals were infested and recaptured twice and thrice, respectively. Recapture rates of the brood-rearing adult birds were high (> 79%) in the two breeding seasons but considerably lower (*c.*50%) in the winter season (Table 1).

**Table 1:** The number of great tits experimentally infested with *I. ricinus* larvae and recaptured in the wild per season.

	No. of infested birds	No. of recaptured birds	Recapture success (%)
Breeding 2022	69	65	94.2
Summer/Autumn 2023	62	NA	NA
Winter 2022–2023	169	79	46.7
Breeding 2023	98	78	79.6

*Notes*: In both breeding seasons, birds were (re)captured in the nestboxes while feeding their chicks. In winter, birds were (re)captured in the nestboxes while roosting. During Summer/Autumn 2022, 62 birds were caught in the wild but kept in captivity during xenodiagnosis, thus there was no recapturing.

*Abbreviation*: NA, not applicable.

For both breeding seasons, we also checked whether nest failure rates (between initial capture of parents and fledging) were higher for nests with xenodiagnosed parents than control nests in the same population (first broods only) where at least one parent was captured but no xenodiagnosis performed. In both years, nest failure rates of nests with xenodiagnosed parents (X) were even lower than control (C) nests (2022: X_(*n* = 39)_ = 0%, C_(*n* = 128)_ = 6.2%; 2023: X_(*n* = 50)_ = 10%, C_(*n* = 124)_ = 14.5%), suggesting a minimal impact of our methodology on the breeding success of the infested individuals.

### 3.2 Tick attachment and recovery

Before infestation, both nymphs and larvae of wild origin were found on the birds, but in lower numbers compared to the number of larvae removed after xenodiagnosis, particularly in summer/autumn. Even in the two breeding seasons, the mean number of wild larvae was only around two per bird (Table 2).

**Table 2:** The mean number of wild ticks (larvae and nymphs) counted on the great tits pre-infestation per season, and outcomes of the infestation with pathogen-free larvae.

Season	Mean no. of wild ticks pre-infestation ± SD	No. of infested larvae	Mean no. of larvae not attached to bird ± SD	Mean no. of recovered larvae ± SD
Breeding 2022	Larvae: 2.28 ± 3.09	40	1.76 ± 1.78 (*n* = 69)	13.89 ± 7.87 (*n* = 38)
	Nymphs: 5.18 ± 4.27 (*n* = 69)			
Summer/Autumn 2022	Larvae: 0.7 ± 1.43	40	1.05 ± 1.30 (*n* = 62)	13.58 ± 5.78 (*n* = 62)
	Nymphs: 0.07 ± 0.31 (*n* = 62)			
Winter 2022–2023	NA	40	NA	8.47 ± 6.87 (*n* = 79)
Breeding 2023	Larvae: 1.61 ± 2.19	40	0.70 ± 1.30 (*n* = 98)	19.17 ± 12.35 (*n* = 78)
	Nymphs: 2.87 ± 3.15 (*n* = 98)			

*Notes*: The last column includes experimentally infested larvae as well as wild larvae. During the winter season, wild ticks were not counted pre-infestation. Except for the winter season, all birds were kept in bags for 1 h and the number of unattached larvae were counted in the bags, after releasing the birds. The great tits were recaptured after 2 days (both breeding and Summer/Autumn season) and 2.5 days (Winter season), the birds recaptured after 3 days in breeding season 2022 were not included in this column.

*Abbreviation*: *n*, number of birds; SD, standard deviation; NA, not applicable.

During the first breeding season, we initially recaptured the birds after 3 days, resulting in a low recovery rate of 4.0 larval ticks recovered per bird (SD = 3.3, *n* = 27). We hypothesized that this was due to larvae detaching earlier than we had anticipated, and therefore we started recapturing after 2 days, which significantly increased the number of recovered ticks to 13.89 larvae per bird (SD = 7.87, *n* = 38) (*t* = 7.07, *df* = 60.67, *P* < 0.01).

Overall, we found a significant difference in the number of recovered ticks between seasons (Kruskal-Wallis test, *χ*^2^ = 49.16, *df* = 3, *P* < 0.01). Recovery of larvae was highest in the breeding season of 2023 followed by the breeding season of 2022 and summer/autumn 2022, and lowest in winter 2022–2023 (Table 2). Dunnʼs test indicated that only the winter season was significantly different from the other seasons, while no significant difference was found between the other seasons (all *P* > 0.2).

### 3.3 *Borrelia* screening outcomes

**Fig. 2:**
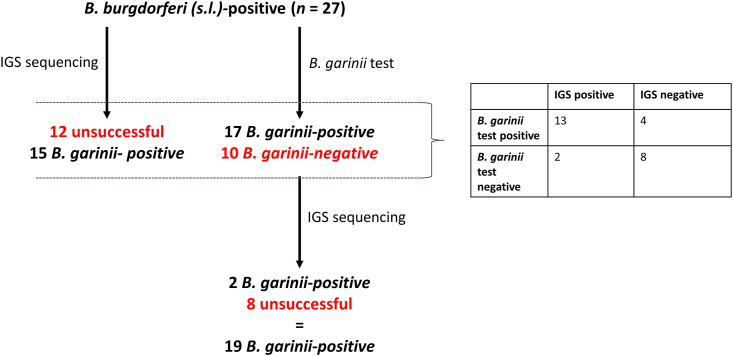
Overview of outcomes of the first breeding season for 27 larvae that were found positive for *Borrelia burgdorferi* (*s.l*.) and subsequently tested (i) by sequencing the 5S-23S intergenic spacer region (IGS) and (ii) running the *B. garinii*-specific qPCR. The figure compares the conventional approach, where *B. garinii* was detected by IGS sequencing, with the new approach, which combines our newly developed *B. garinii* qPCR and IGS sequencing. The contingency table summarizes the same information.

The larvae of the first breeding season that tested positive with the *B. burgdorferi* (*s.l.*) qPCR were all tested with the *B. garinii-*qPCR and IGS-sequencing (*n* = 27). Traditionally, larvae that tested positive for *B. burgdorferi* (*s.l*.) on the qPCR would be Sanger sequenced to determine the *B. burgdorferi* (*s.l.*) genospecies. This approach resulted in 15 *B. garinii*-positive larvae, while in 12 larvae we were not able to determine the presence of a *Borrelia* genospecies due to unsuccessful sequencing (Fig. 2). No other genospecies were detected. Seventeen of 27 larvae tested positive on the *B. garinii-*qPCR, whereas 13 tested positive for *B. garinii* with the traditional approach. The remaining 10 larvae were sequenced, adding 2 extra *B. garinii-*positive larvae to the total (Fig. 2). Thus, by adding the *B. garinii*-specific qPCR to the conventional approach we were able to identify 4 additional *B. garinii*-positive larvae. Moreover, we only had to sequence 10 larvae instead of 27, drastically reducing the workload and cost. Finally, with the new approach, only 8 larvae did not give conclusive outcomes (positive or negative) compared to 12 with the traditional approach.

Given the advantages of including the *B. garinii*-specific qPCR, we decided to use this method for the larval ticks from the other seasons. In the following seasons, we found that of the 3006 larvae, 613 larvae tested positive for *B. burgdorferi* (*s.l.*), of which 521 larvae tested positive with the *B. garinii*-qPCR. After Sanger sequencing, among the larvae that tested negative on the *B. garinii-*qPCR (*n* = 92), we found 39 additional *B. garinii-*positive larvae, 36 larvae that tested positive for another *Borrelia* genospecies (13 *B. turdii* and 23 *B. valaisiana*) and 17 cases where the sequencing was unsuccessful. Again, we detected most positive larvae with the *B. garinii*-specific test and reduced the amount of Sanger sequencing by 85%.

### 3.4 Birds with *Borrelia garinii*-positive larvae

Out of the 257 successfully conducted xenodiagnoses (excluding recaptures after 3 days), 49 birds had at least one *B. garinii*-positive larva. The proportion of birds with positive larvae varied greatly between seasons (*χ*^2^ = 64.41, *df* = 3, *P* < 0.01). In the breeding season of 2023, 49% of birds had at least one *B. garinii*-positive larva, while in the other seasons, the percentage of birds with at least one positive larva was not greater than 11% (Table 3). The *post-hoc* analysis indicated that the breeding season of 2023 differed significantly from all other seasons (all *P* < 0.01), while no significant difference was found between the other seasons (all *P* = 1.00). This was also reflected in the overall rate of positive larvae which was 35.7% in the breeding season of 2023, and less than 3% in all other seasons.

**Table 3:** *Borrelia garinii* presence in the screened larvae collected from great tits that successfully underwent xenodiagnosis in each season.

	Breeding 2022	Summer/Autumn 2022	Winter 2022–2023	Breeding 2023
No. of screened larvae (No. of infested birds, *n*)	528 (*n* = 38)	842 (*n* = 62)	669 (*n* = 79)	1495 (*n* = 78)
No. of *B. garinii-*positive larvae (No. birds with positive larvae, *n*)	13 (*n* = 4)	8 (*n* = 3)	18 (*n* = 4)	534 (*n* = 38)
Mean no. of positive larvae of birds with at least one positive larva ± SD	3.2 ± 3.9	2.7 ± 2.1	4.5 ± 7.0	14.1 ± 11.1
Prevalence of *B. garinii*-infected larvae (Prevalence of infected birds)	2.5% (11%)	0.9% (5%)	2.7% (5%)	35.7% (49%)

*Abbreviation*: SD, standard deviation.

Xenodiagnoses were conducted twice on 33 individuals, excluding recaptures after 3 days. Most of these individuals were non-infectious in all xenodiagnoses (*n* = 28), while a few were only infectious in one xenodiagnosis (*n* = 5). Additionally, one individual was infectious in two xenodiagnoses and negative in a third test, the latter however with only two larvae, and another individual was not infectious thrice.

**Fig. 3:**
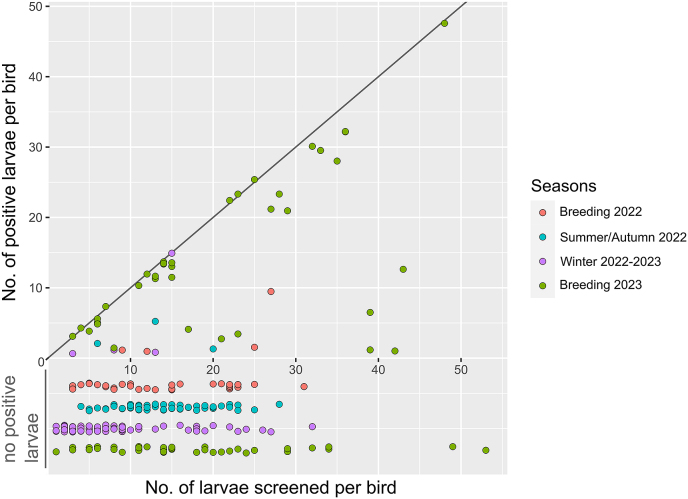
The number of *B. garinii*-positive larvae *versus* the number of screened larvae for each experimentally-infested bird over the four seasons. Each dot represents one xenodiagnosed great tit. To increase clarity, individual birds with zero positive larvae are shown below the horizontal axis per season, while birds with at least one positive larva are shown above the horizontal axis. The diagonal line indicates a 100% positivity rate of the recovered larvae.

Birds with at least one positive larva showed considerable variation in their infectiousness. In the breeding season of 2023, a large number of birds had all or nearly all larvae positive for *B. garinii* (shown as data points close to the 1:1 line in Fig. 3), while a smaller number of individuals had a low fraction of positive larvae. In the other seasons, the results were opposite, with most birds showing low infectiousness, with one exceptional case in the winter of 2022–2023 (Fig. 3).

## 4 Discussion

Tick-borne infection studies in the wild generally focus on the variation in competence of potential reservoir hosts for an infection, while variation within species remains largely understudied (Brunner et al., 2008; Råberg, 2012). Moreover, very few studies in the wild have focused on the question when and how vectors are infected by hosts. Xenodiagnoses makes it possible to get a better insight in those understudied components of the ecology of Lyme borreliosis (Michigan et al., 2012; Wolcott et al., 2021; Norte et al., 2022). Using xenodiagnosis in the wild, for which timely recapture of the birds was essential, we were able to successfully gather information about the infectiousness of great tits in different seasons and with large sample sizes. To our knowledge, we conducted the first direct xenodiagnoses in the wild on tick-borne infections on targeted individual hosts, as well as for vector-borne diseases on free-living hosts during the xenodiagnoses. Previous studies on wild animals have kept the animals in captivity, sometimes under anaesthesia, for the duration of the xenodiagnosis (Molina et al., 2012; Jiménez et al., 2014; Marinho-Júnior et al., 2023).

Applying xenodiagnosis in free-living host populations for estimating variation in infectiousness requires large sample sizes, which poses various challenges. First, a substantial number of birds must be recaptured post-infestation, and sufficient larvae should be recovered from the birds. Secondly, efficient screening of the larvae for *B. garinii* is crucial. We found that recapture rates of the infested birds were very high during both breeding seasons (94.2% and 79.6%, respectively in 2022 and 2023). These recapture rates offer perspectives for routine application of xenodiagnoses at individual level with minimal risk of biases due to variation in trappability (Kidd et al., 2015). In contrast to the breeding seasons, we had a lower recapture rate in the winter of 2022, where only half of the individuals were recaptured. Presumably, individuals that were not recaptured did not roost in nestboxes, but instead made use of natural cavities. Previous data showed that disturbance during roosting decreases recapture success (Tyller et al., 2012; Matthysen, unpublished data). The lower recapture rate implies that xenodiagnosis outcomes in winter may be biased by trappability, which in turn may be related to individual variation in behaviour, infection status, or parasite loads (Garamszegi et al., 2009; Boyer et al., 2010; Holmes et al., 2024). Our study suggests that our methodology can be applied to any host that can be captured and recaptured, before the vector completes its blood meal, in a targeted way within and also across seasons. This includes most breeding birds, especially those breeding in easily accessible nests, such as nestbox-using birds (including some widely studied model organisms in ecology) or colonial nesting seabirds. Similarly, species that can be frequently recaptured at feeding or resting sites could also be studied this way outside the breeding season. In addition, vectors with short feeding durations (e.g. mosquitoes) cannot be used for xenodiagnoses on free-living animals. These xenodiagnoses need to be conducted in captive conditions, depriving hosts of their natural activities and causing additional stress, which may influence infectiousness. While our approach may likely be biased towards a particular subset of species and environments, it nevertheless creates the potential for significant advancements in understanding the ecology of wildlife diseases and zoonoses in the wild.

Besides the recapture success of the host, an important success factor for xenodiagnosis is whether sufficient ticks are recovered. This allows the detection of *Borrelia* infections even in hosts that only transmit *Borrelia* to a low number of ticks. In addition, high tick recovery makes it possible to estimate the degree of infectiousness of individuals more precisely. Since we found, on average, low numbers of larvae of wild origin on the birds (Table 2), experimentally infesting the birds with larvae is important. By experimentally infesting the birds, we also eliminate a possible bias of only having results from birds with larger numbers of wild larvae, thus creating a possible confounding between infectiousness and tick burden at the time of xenodiagnosis.

We found large seasonal variation in the ability of birds to transmit *B. garinii* to feeding *I. ricinus* larvae (i.e. variation in infectiousness). In the breeding season of 2023, most birds had high infectiousness, and a smaller fraction of birds had low infectiousness. In the other seasons, by contrast, most of the birds had low infectiousness. While the former group can clearly be considered highly infectious, for birds that had a single positive larva out of many, limited vector-to-vector transmission through co-feeding is a possible explanation (Voordouw, 2015). Under co-feeding transmission, a *B. garinii*-positive nymph of wild origin would have transmitted *B. garinii* to a neighbouring larva. A previous study showed a small number of *B. afzelli-*positive larvae on great tits, demonstrating co-feeding transmission (Heylen et al., 2017b). We therefore cannot be certain whether a single larva was infected by a bird with low infectiousness or *via* co-feeding with a neighbouring *B. garinii*-infected wild nymph. Another possibility is that we underestimated the infectiousness of all birds, since the larvae did not entirely complete their blood meal. A longer attachment of the larvae increases the intake of *Borrelia* spirochetes, as has been shown with *I. scapularis* larvae and *B. burgdorferi* (*s.s.*) (Couret et al., 2017). Nevertheless, we found many birds with (nearly) all larvae positive, indicating that our method can detect high levels of infectiousness.

We recovered high proportions of xenodiagnostic larvae during the breeding seasons and in summer/autumn, in comparison to winter. The lower tick recovery was likely related to the lower temperatures in winter, which is known to decrease tick activity and likely larval attachment/feeding success (Heylen et al., 2014c; Kahl and Gray, 2023). In addition to the less-than-optimal temperatures in winter for larvae, tick attachment may also have been affected by the infestation protocol. In both the breeding and summer/autumn seasons, we put the birds in a cotton bag post-infestation, increasing the likelihood of tick attachment. In contrast, during winter, we did not put the birds in cotton bags, which may have decreased tick attachment success and subsequent tick recovery. On average and over all seasons, over half of the number of larvae were not recovered. Grooming behaviour of the birds could have contributed to the loss of larvae. In principle, the number of recovered larvae could be enhanced by infesting birds with higher larval tick load. However, out of precaution, we chose to infest the birds with only 40 larvae as higher numbers of ticks can reduce the health status of the birds. Previous studies showed that infesting great tits with *I. ricinus* nymphs and adults had measurable but minor effects on the health status of adult birds (Heylen and Matthysen, 2008; Heylen et al., 2010), but no effects were found of *I. ricinus* larval ticks on the health status of great tit nestlings (Heylen and Matthysen, 2011). The limited harm, due to the loss of erythrocytes, was only short-term and had no effect on the general body condition of the birds (Heylen et al., 2010). We decided to infest the birds with a number of larvae within the natural range, nonetheless, we had no control over the number of wild ticks prior as well as during the xenodiagnoses (Heylen et al., 2014c).

We could not distinguish between laboratory-reared and wild larvae collected from the birds. Despite the wild origin of these larvae, these are equally informative as vertical transmission of *B. garinii* is likely very rare (Heylen et al., 2014a; Voordouw, 2015). As the great tit was their first blood meal, it was also their first opportunity to acquire any *Borrelia* species. Comparing the number of wild larvae pre-infestation to the number of larvae collected from the recaptured birds showed that the wild larvae accounted for a small percentage of the total number of collected larvae (Table 2). In winter, we did not count ticks pre-infestation, but the number of wild larvae was presumably very low due to low tick activity (Heylen et al., 2014c). In summer/autumn, when conducting xenodiagnoses in the laboratory, the number of wild larvae among the recovered larvae was likely even less since no new larvae could attach during xenodiagnosis.

An essential aspect of our methodology involves successfully screening larval ticks for *B. garinii*. Previous studies have shown that the success of IGS-based genotyping can be highly variable (Hamšíková et al., 2017; Coipan et al., 2018). In addition, Sanger sequencing is time intensive and costly. Therefore, we developed a *B. garinii-*qPCR to reduce the number of false negatives and the amount of Sanger sequencing needed. We found that we could successfully screen most *B. burgdorferi* (*s.l.*)-positive larvae with our *B. garinii-*qPCR thereby drastically reducing the number of larvae that had to be Sanger sequenced. Furthermore, we detected more *B. garinii-*positive larvae by adding the *B. garinii-*qPCR before Sanger sequencing (see Fig. 2). In ticks co-infected with multiple genospecies of *B. burgdorferi* (*s.l.*), the *B. garinii*-qPCR also makes it possible to detect *B. garinii* even when it is the less abundant *Borrelia* genospecies in the tick. In contrast, Sanger sequencing of amplicons targeting all *B. burgdorferi* (*s.l.*) genospecies tends to identify only the most abundant genospecies (Mysterud et al., 2019a). We conclude that the addition of our *B. garinii*-specific qPCR to the conventional approach makes detection of *B. garinii* more sensitive, faster and cost-effective.

Our preliminary results underline the importance of studies in the wild focusing on within species variation of infectiousness not only between seasons, but also between years. We found a remarkably higher proportion of birds with positive larvae during the breeding season of 2023 than in the other seasons. Not only were more birds found to be infectious, but also most birds had a high infectiousness. Potential explanations for the difference between the two breeding seasons could include changes in the density of infected nymphs (DIN), changes in maternally inherited immunity, or changes in age structure of the great tit population (Gasparini et al., 2001; Heylen et al., 2013; Bregnard et al., 2020). Although the average number of nymphs counted on the birds in the breeding season of 2023 was lower than in 2022, more birds were infectious in 2023. Infection prevalence in nymphs taken from the birds was also much higher in 2023 than in 2022 (our unpublished data), similar to the larvae, but we cannot infer whether these nymphs were infected in the larval or nymphal stage. We need more research to unravel the causes of this remarkable variation in infectiousness in our great tit populations. So far, only few studies have investigated temporal variation in *Borrelia* infectiousness of a wild host. Previous research on *B. burgdorferi* (*s.l.*) in wild bank voles has also shown temporal variation in infectiousness to feeding *I. ricinus* ticks (Tälleklint et al., 1993; Tälleklint and Jaenson, 1995). Interestingly, in their study infectiousness was higher in August to September while great tits, based on our preliminary results, seem to have higher infectiousness during the breeding season. To further investigate this variation, we aim to combine repeated xenodiagnoses of the same individuals over time, analyzing individual characteristics and life history of each bird. To obtain an in-depth understanding of the temporal changes in infectiousness, more data also needs to be collected throughout all seasons, making it possible to further disentangle seasonal and yearly effects.

## 5 Conclusions

We successfully employed a methodology to quantify infectiousness of tick-borne pathogens in free-living great tits. This makes us possibly the first to quantify infectiousness in individual free-living animals for any vector-borne disease. With this method we were able to recapture large numbers of birds, especially in the breeding season. These recaptured birds had higher larval tick numbers during the breeding seasons than in the winter. We successfully screened those larvae for *B. garinii* with our novel *B. garinii*-specific qPCR, which is more sensitive, cheaper, and faster than the traditional approach. The results of the methodology showed a large seasonal variation in infectiousness, which will be investigated further.

## Funding

This study was funded by FWO-10.13039/501100011878Flanders (grant G052422N) to Erik Matthysen, Dieter Heylen and Hein Sprong.

## Ethical approval

The study protocol was approved by the Ethics Committee for Animal Testing of the University of Antwerp (2022–02). Bird capturing and handling was permitted by a ringing licence of the Flemish Ministry, and an exemption for research on protected species (FF V22-00110).

## Data availability

The data supporting the conclusions of this article are included within the article and its supplementary file.

## CRediT authorship contribution statement

**Jens Zarka:** Writing – review & editing, Writing – original draft, Visualization, Methodology, Investigation, Formal analysis, Data curation, Conceptualization. **Dieter Heylen:** Writing – review & editing, Supervision, Methodology, Funding acquisition, Conceptualization. **Hein Sprong:** Writing – review & editing, Supervision, Methodology, Funding acquisition, Conceptualization. **Manoj Fonville:** Methodology, Investigation, Data curation. **Joris Elst:** Investigation, Methodology. **Erik Matthysen:** Writing – review & editing, Supervision, Methodology, Funding acquisition, Conceptualization.

## Declaration of competing interests

The authors declare that they have no known competing financial interests or personal relationships that could have appeared to influence the work reported in this paper.

## Data Availability

The data supporting the conclusions of this article are included within the article and its supplementary file.

## Appendix A Supplementary data

Supplementary Table S1 Raw data. Supplementary Table S1
